# Nuclear Calcium in Cardiac (Patho)Physiology: Small Compartment, Big Impact

**DOI:** 10.3390/biomedicines11030960

**Published:** 2023-03-21

**Authors:** Mara Kiessling, Nataša Djalinac, Julia Voglhuber, Senka Ljubojevic-Holzer

**Affiliations:** 1 Department of Cardiology, Medical University of Graz, 8036 Graz, Austria; 2 Department of Biology, University of Padua, 35122 Padova, Italy; 3 BioTechMed Graz, 8010 Graz, Austria; 4 Gottfried Schatz Research Center, Division of Molecular Biology and Biochemistry, Medical University of Graz, 8010 Graz, Austria

**Keywords:** nuclear calcium, excitation-contraction coupling, excitation-transcription coupling, cardiac remodeling, heart failure, atrial fibrillation

## Abstract

The nucleus of a cardiomyocyte has been increasingly recognized as a morphologically distinct and partially independent calcium (Ca^2+^) signaling microdomain, with its own Ca^2+^-regulatory mechanisms and important effects on cardiac gene expression. In this review, we (1) provide a comprehensive overview of the current state of research on the dynamics and regulation of nuclear Ca^2+^ signaling in cardiomyocytes, (2) address the role of nuclear Ca^2+^ in the development and progression of cardiac pathologies, such as heart failure and atrial fibrillation, and (3) discuss novel aspects of experimental methods to investigate nuclear Ca^2+^ handling and its downstream effects in the heart. Finally, we highlight current challenges and limitations and recommend future directions for addressing key open questions.

## 1. Introduction

Calcium (Ca^2+^) is a ubiquitous intracellular second messenger regulating a plethora of intricate cellular events and functions, e.g., electrical signaling, contraction, secretion, gene transcription and cell cycle [1]. In the heart, Ca^2+^ most prominently mediates the translation of electrical stimulation into the mechanical activity of cardiomyocytes—also known as excitation-contraction coupling (ECC)—thus inducing the contraction of cardiac muscle [2]. Each heartbeat is characterized by a precisely regulated transient rise in cytoplasmic free Ca^2+^ to systolic levels of about 1 µM at peak contraction and subsequent removal to diastolic levels of about 100 nM at full relaxation [3,4]. Beyond this well-described role in cardiomyocyte contractile function, Ca^2+^ has emerged as a key player in both physiological and pathophysiological cardiac signaling cascades and the regulation of gene transcription. It can thereby not only exert acute cellular effects but also contribute to long-term changes through so-called excitation-transcription coupling (ETC) [5,6,7].

Considering that the entire cardiomyocyte’s cytosol is flooded with Ca^2+^ on a beat-to-beat basis, there is an obvious need for the cell to spatio-temporally distinguish between “contractile” and “signaling” Ca^2+^ to properly elicit specific cellular functions [8]. This is in part achieved by the subcellular organization of cardiomyocytes in so-called microdomains that permit targeted Ca^2+^ release and thereby facilitate locally restricted Ca^2+^ signaling events [9,10]. The nucleus is considered as such distinct microdomain harboring its own nucleoplasmic Ca^2+^ transient cycling machinery and signaling properties with important implications for the regulation of gene transcription [7,10,11]. While alterations in cytoplasmic Ca^2+^ handling have been extensively studied and associated with the pathogenesis of cardiac remodeling, heart failure (HF) and atrial fibrillation (AF); nucleoplasmic Ca^2+^ has received considerably less attention. In this review, we will discuss the role of nuclear Ca^2+^ in cardiac physiology and pathophysiology and summarize recent findings on nuclear Ca^2+^ calibration methods and imaging tools. We will assess new developments in experimental approaches and highlight yet unanswered questions to stimulate further research.

## 2. Nuclear Ca^2+^ Dynamics

### 2.1. Structural Basis of Nuclear Ca^2+^ Handling

The nucleus is a subcellular compartment separated from the cytoplasm by the nuclear envelope (NE). Consisting of two phospholipid bilayers, the NE acts as a barrier and selective filter for the trafficking of substances and molecules in and out of the nucleus. Throughout the NE, nuclear pore complexes (NPCs) facilitate the diffusion of ions and small molecules (5 nm and 40–60 kDa) [12,13] and actively shuttle bigger proteins and RNAs (≤39 nm) via an associated transport system [14]. The inner and outer nuclear membranes show distinct characteristics determined by their orientation and the adjacent compartment. The outer nuclear membrane faces the cytoplasm and transitions directly into the membrane of the sarcoplasmic reticulum (SR), whereas the inner nuclear membrane is directed towards the nucleoplasm forming deep invaginations into the nuclear lumen, referred to as the nuclear reticulum [15,16]. Various Ca^2+^ release and re-uptake proteins and their regulators are embedded in the NE, such as inositol-1,4,5-triphosphate receptors (IP_3_Rs) [17], as well as the SR Ca^2+^ ATPase (SERCA) [18], phospholamban (PLB) [19] and the sodium-calcium exchanger (NCX) [20], highlighting the nucleus as an autonomous, fully-equipped Ca^2+^ handling compartment. The area between both membranes is similar in ion and protein composition to the SR and acts as a local Ca^2+^ storage [15]. Deep T-tubules are frequently located in very close proximity to the NE forming local Ca^2+^ signaling hubs, often accompanied by prominent mitochondrial accumulation [21,22,23].

### 2.2. Nuclear Ca^2+^ Transients

**Figure 1:**
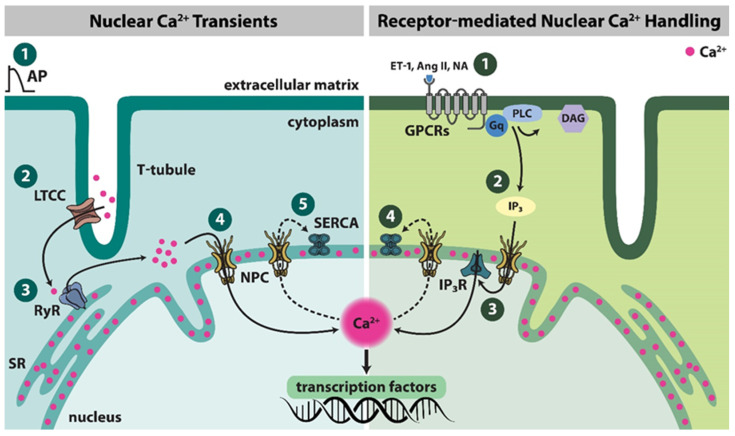
Nuclear Ca^2+^ handling in cardiomyocytes. (**Left**) Cardiac action potential (AP) causes myocyte membrane depolarization (1) and creates an inward Ca^2+^ current through opening of L-type Ca^2+^ channels (LTCC) located at the plasma membrane, including T-tubules (2). Increased local Ca^2+^ concentration ([Ca^2+^]) triggers the release of Ca^2+^ stored in the sarcoplasmic reticulum (SR) via ryanodine receptors (RyR) (3). Nuclear pore complexes (NPC) facilitate passive diffusion of free cytoplasmic Ca^2+^ into the nucleus (4), where the transient rise in [Ca^2+^]_nuc_ can mediate transcriptional effects. For [Ca^2+^]_nuc_ to decline, Ca^2+^ has to diffuse back out of the nucleus through NPCs to be taken up into the nuclear envelope by SR Ca^2+^ ATPase (SERCA) expressed on the outer nuclear membrane (5). (**Right**) G-protein coupled receptors (GPCRs) are stimulated by endothelin-1 (ET-1), angiotensin II (Ang II) or noradrenaline (NA) and activate phospholipase C (PLC) (1), which synthesizes diacylglycerol (DAG) and inositol-1,4,5-triphospate (IP_3_) (2). IP_3_ then passively diffuses through NPCs to initiate Ca^2+^ release from the nuclear envelope via IP_3_ receptors (IP_3_R) and thus elicits a nuclear Ca^2+^ release (3). Increased [Ca^2+^]_nuc_ can again exert effects on transcription factors before diffusing back out of the nucleoplasm via NPCs and being taken up by SERCA expressed on the outer nuclear membrane (4).

Every cytosolic Ca^2+^ transient (CaT) in a cardiomyocyte elicits a nuclear CaT (Figure 1, *left*). This also means that any change in cytoplasmic Ca^2+^ concentration, [Ca^2+^]_cyto_, e.g., following neurohormonal stimulation, will be accompanied by changes in nucleoplasmic Ca^2+^ concentration, [Ca^2+^]_nuc_ [24]. Nuclear CaTs are characterized by a slower and delayed upstroke, lower peak and prolonged decay time in comparison to cytosolic CaTs. The delayed upstroke is likely caused by the insulation of the nucleus by the NE and the slower spread of Ca^2+^ released from the SR through a limited space within the NPCs, introducing a kinetic delay and simultaneously reducing the amplitude [25]. This idea is supported by the documented absence of major Ca^2+^ release channels from the NE, such as ryanodine receptors (RyRs), which are instead found in close proximity to the NE, forming a “cage” around the nucleus [26,27,28]. Similarly, [Ca^2+^] decline is mediated by passive diffusion out of the nucleus through NPCs and subsequent SR/NE SERCA reuptake or extrusion via NCX located in nearby T-tubules. As the large majority of SERCA is located on the outer nuclear membrane, Ca^2+^ first needs to diffuse out of the nucleus through NPCs to be taken up again into the lumen of the NE [24,29]. In a series of elegant experiments in which they modulated SERCA function with thapsigargin, Kiess and Kockskaemper indeed confirmed that—together with cytoplasmic Ca^2+^ levels—SERCA function is the single most important factor regulating diastolic [Ca^2+^]_nuc_ [24]. Although the NPCs always remain open, their conductance and thereby total Ca^2+^ diffusion capacity can vary, depending on intracellular [Ca^2+^] and [ATP] [30], as well as distribution, density and position of NPCs on the NE [31]. Due to these structural and functional features, changes in stimulation frequency differentially affect nuclear and cytosolic diastolic CaTs. Whereas systolic [Ca^2+^]_nuc_ increases to a similar extent as [Ca^2+^]_cyto_ in response to faster pacing, diastolic [Ca^2+^]_nuc_ rises to about twice the [Ca^2+^]_cyto_, suggesting that slower nucleoplasmic CaT kinetics provoke a build-up of Ca^2+^ when diastole is shortened [32]. If prolonged, such diastolic [Ca^2+^]_nuc_ overload may lead to the activation of Ca^2+^-mediated hypertrophic pathways in the nucleus with important structural and functional consequences for the heart.

Recent work uncovered an additional regulatory mechanism of nuclear CaTs in cardiomyocytes, which prevents nucleoplasmic Ca^2+^ overload, especially in conditions of physiological stress. Namely, Voglhuber and Holzer et al. found that in healthy cardiomyocytes, densely packed mitochondria in perinuclear spaces—small areas of cytoplasm adjacent to the nucleus in the longitudinal direction—shape nucleoplasmic CaTs by taking up significant amounts of Ca^2+^ in close proximity to the nucleus. In contrast, pharmacological inhibition of mitochondrial Ca^2+^ uptake led to a significant increase in Ca^2+^ levels in and around the nucleus [21].

### 2.3. Nuclear Ca^2+^ Sparks and Puffs

As a result of the close proximity of NE and SR, the nucleus reacts to spontaneous, local Ca^2+^ release events from RyR2s and IP_3_Rs—so-called Ca^2+^ sparks and puffs—with a brief increase in [Ca^2+^]_nuc_ independent of [Ca^2+^]_cyto_ [33,34]. Similarly, the small distance between the NE and T-tubules and dyads regulates nuclear Ca^2+^ content without influencing global [Ca^2+^] [27]. PLB, the endogenous inhibitor of SERCA, is highly concentrated in the NE with a greater PLB to SERCA ratio than in the SR and plays a key role in the regulation of such localized nuclear Ca^2+^ dynamics [19,35]. Indeed, modulation of PLB activity affects the amount of Ca^2+^ taken up into lumen of the NE and regulates IP_3_R-RyR2 mediated spontaneous Ca^2+^ release [34]. As levels and activity of IP_3_R and PLB dramatically change during (patho)physiological cardiac stress [25,36], the altered dynamics of nuclear Ca^2+^ sparks and puffs are likely causally involved in the progression of adverse cardiac remodeling.

### 2.4. Receptor-Mediated Nuclear Ca^2+^ Handling

In addition to its regulation via passive diffusion of Ca^2+^ from the cytoplasm or specialized microdomains such as dyads, [Ca^2+^]_nuc_ can be additionally and independently regulated via the direct release of Ca^2+^ from the NE [37]. Such active regulation of nuclear Ca^2+^ levels involves different classes of receptors located on and in close proximity to the NE.

#### 2.4.1. IP_3_R

Key components of autonomous nuclear Ca^2+^ regulation processes are IP_3_Rs, with IP_3_R type 2 (IP_3_R2) expressed mainly in ventricular [26,36] and IP_3_R type 1 (IP_3_R1) in atrial cardiomyocytes [38], though recent evidence suggests the possibility of all IP_3_R subtypes (1–3) being present in both neonatal and adult cardiomyocytes [39]. IP_3_Rs are activated following G-protein coupled receptor (GPCR)-dependent activation of phospholipase C and generation of IP_3_ which then binds to the IP_3_Rs, instigating Ca^2+^ release from intracellular Ca^2+^ stores (Figure 1, *right*). Examples of GPCRs include endothelin-1 (ET-1), angiotensin-II (Ang II) and insulin-like growth factor 1 (IGF-1) receptors [40,41,42], which once coupled to their agonists are implicated in promoting hypertrophy. Cardiomyocytes show a unique pattern of IP_3_R distribution, with evidence that they are concentrated in nuclear and perinuclear compartments [26,28], thereby facilitating Ca^2+^-mediated transcriptional changes. The distribution of IP_3_Rs can, however, differ depending on the onset, intensity and duration of nuclear IP_3_-mediated Ca^2+^ signaling and finely tune activation of its downstream targets, such as Ca^2+^/calmodulin-dependent protein kinase II (CaMKII) and calcineurin (CaN) [26,28,40,43,44].

While IP_3_R-dependent regulation of [Ca^2+^]_nuc_ is consistently shown to be mediated by Ca^2+^ release from perinuclear stores, precaution is needed when choosing an appropriate model to study IP_3_R signaling, since it is dependent on specific upstream stimuli, cardiomyocyte species and their maturation stage [32,40,45,46,47,48,49]. Ang II acts through IP_3_Rs to over-proportionally raise Ca^2+^ in the nucleus versus cytosol, both at rest [44] or when cells are paced at baseline frequencies [28]. This increase in [Ca^2+^]_nuc_ is further sensed by CaN, which activates nuclear factor of activated T cells (NFAT)-dependent transcription [44]. Similarly, IGF-1 is also capable of eliciting an IP_3_R-mediated increase in nuclear Ca^2+^ levels. IGF-1 was shown to induce higher peak amplitudes and larger CaTs in the nucleus, which were clearly attributable to Ca^2+^ release via IP_3_Rs, as demonstrated using IP_3_R and RyR2 inhibitors [50]. This may be facilitated by the existence of a locally restricted signaling toolkit formed by deep T-tubular invaginations towards the NE, harboring the IGF-1 receptor and providing spatial insulation from whole cell Ca^2+^ oscillations [42]. Nakao et al. further suggested the involvement of neuronal calcium sensor-1 (NCS-1)—known for its pivotal role in various neuronal functions—in regulating nuclear and cytoplasmic Ca^2+^ signals mediated by IGF-1 in cardiomyocytes. In their study, NCS-1 co-localized with IP_3_Rs on the NE and in perinuclear regions [50]. This is in line with a previous report on the interaction between NCS-1 and IP_3_Rs as an important regulator of hypertrophy, engaging both CaMKII and CaN pathways [51].

#### 2.4.2. Adrenergic Receptors

Both in vivo and in vitro studies have demonstrated nuclear and perinuclear localization of α_1_-adrenergic receptors and their signaling partners in cardiomyocytes [52,53,54,55,56]. This is in contrast to the conventional notion that GPCRs localize to and signal at the plasma membrane [57,58,59]. Mechanistically, evidence suggests a α_1_A-subtype/PKCδ/cTnI mediated “inside-out” (nuclear-to-cytoplasmic) signaling pathway, describing the transport of signals initiated at α_1_-ARs in the inner nuclear membrane to cytosolic (sarcomere) or membrane targets where the effects on cardiomyocyte contractile function are then elicited [52]. In line with this, α_1_-AR agonist, phenylephrine (PE), induced an increase in IP_3_R-mediated nuclear CaT frequency and triggered Ca^2+^-induced Ca^2+^ release in the cytosol of isolated neonatal rat cardiomyocytes [47]. However, insights into other contractile regulators controlled by nuclear α1-ARs are still sparse due to the limitations in techniques to detect α_1_-ARs [60]. Several studies including patient datasets have verified a cardioprotective role of α_1_-AR activation [52], thus emphasizing the therapeutic potential of modulating nuclear α-AR signaling.

Conversely, β-AR expression on cardiomyocyte nuclei is more controversially discussed. On the one hand, Boivin et al. demonstrated by immunological, ligand-binding and functional criteria that in rat and mouse adult ventricular cardiomyocytes β_1_AR- and β_3_AR-subtypes are located on the nuclear membrane [61]. Similarly, downstream effectors such as adenylyl cyclase (AC) and protein kinase A (PKA) were shown to be associated with the nucleus or the nuclear membrane [62], and β_1_AR stimulation with isoproterenol resulted in both increased AC activity and modulated gene expression levels in isolated nuclei from rat hearts [61,63]. Wang et al. further observed β_1_AR expression at the SR, associated with SERCA and PLB, to promote the phosphorylation of local targets via PKA activation [64]. On the other hand, Bedioune et al. showed that β_1_ARs and β_2_ARs located in the plasmalemma also differentially regulate nuclear PKA activity upon receptor stimulation, highlighting the formation of signaling routes from the plasmalemma into the nucleus with potential implications on the control of gene expression by βARs [65] without nuclear expression of the receptors. Furthermore, visualization of βARs in cardiomyocytes with highly sensitive microscopy methods failed to show a nuclear specific localization of βARs [66]. Recent evidence suggests the Golgi apparatus as a localized βAR-expression and signaling microdomain in close vicinity to the nucleus [67,68]. Overall, intracellular βAR-signaling emerges as an interesting concept in the regulation of cardiac contractility and may present a novel translational approach in optimizing the subcellular targeting of β-blockers in the future.

Together, the elaborate structural organization of the nucleus and its interplay with other subcellular compartments enable the formation of local, Ca^2+^ sensitive nuclear microdomains. In such a spatially and functionally restricted environment, Ca^2+^ signals can manifest immediate or long-term transcriptional regulatory effects and minutely respond to the current physiological demand of cardiomyocytes.

## 3. Role of Nuclear Ca^2+^ in Cardiomyocytes

Nuclear Ca^2+^ dynamics in cardiomyocytes are mainly associated with the process of ETC, where gene expression is regulated as part of the cellular response to physiological and pathological stress [69,70]. The best-studied signaling cascades activated by increased nuclear Ca^2+^ levels in cardiomyocytes are mediated by CaMKII and CaN. Downstream of CaMKII activation, phosphorylation of histone deacetylase 4 (HDAC4) was shown to promote nuclear HDAC4 export and thereby de-repress pro-hypertrophic transcription factor myocyte enhancer factor 2 (MEF2). MEF2 is involved in cardiac remodeling, fetal gene program re-expression and fibrosis [71,72]. Other transcription factors activated in a CaMKII-dependent manner have also been identified, e.g., the nuclear cyclic adenosine monophosphate (cAMP) response element binding protein (CREB) [73] and nuclear factor-kappa B (NF-κB) [74]. Subedi et al. demonstrated that CaMKII mediates CREB activation in response to ET-1 and PE stimulation in rat ventricular cardiomyocytes [75], while Suetomi et al. identified CaMKII-mediated proinflammatory signaling involving NF-κB upon introduction of pressure overload in mice [74]. Similarly, CaMKII potently represses the expression of L-type Ca^2+^ channels (LTCC) by translocating the downstream regulatory element binding transcription factor DREAM, thus negatively regulating Ca^2+^ influx into the cytosol [76]. Still, it remains an open question whether activation of CaMKII upstream of CREB, NF-κB and DREAM is specifically mediated by nuclear Ca^2+^ accumulation or overall increases in [Ca^2+^] in cardiomyocytes. CaN, as CaMKII, is activated by Ca^2+^/calmodulin and thereby sensitive to elevated [Ca^2+^]. Several factors including differences in association rate with Ca^2+^/calmodulin, regional distribution of CaN, the presence of competing binding partners and endogenous inhibitors allow CaN to evade continuous activation following each contraction [77]. On the other hand, overactivation of CaN leads to pathological gene transcription by targeted phosphorylation of several transcription factors. For instance, CaN dephosphorylates cytosolic transcription factor NFAT and causes its translocation to the nucleus, where it interacts with GATA4 to mediate a hypertrophic response [78]. Importantly, recent work has demonstrated that CaN-NFAT signaling in cardiomyocytes is controlled by perinuclear signalosomes organized by the scaffold protein muscle A-Kinase anchoring protein β (mAKAPβ/AKAP6β) and is sensitive to (peri)nuclear Ca^2+^ [10]. Other major targets of CaN include CRTC, FOXO1 and TFEB [79], yet their specific activation by [Ca^2+^]_nuc_ remains to be addressed.

## 4. Nuclear Ca^2+^ Dysregulation

While alterations in intracellular [Ca^2+^] have generally been accepted as drivers of pathological events in cardiomyocytes, studies reporting isolated changes in nucleoplasmic Ca^2+^ levels are gravely underrepresented in the literature. However, many arguments support the role of [Ca^2+^]_nuc_ as an important regulator of pathological transcription. One strong argument is that functional and structural alterations of the nucleus precede the development of cardiac pathologies. These include changes in nuclear size and shape in the form of NE invaginations and blebbing, together with changes in expression of Ca^2+^ handling proteins, most importantly RyR2s, SERCA and IP_3_Rs [28,44,80,81].

### 4.1. Nuclear Ca^2+^ in Ventricular Remodeling: Hypertrophy to Heart Failure

Cardiac remodeling is characterized by changes in molecular, cellular and interstitial properties of the heart, manifesting as changes in size, shape and function [82]. This includes both physiological and pathological adaptations [83] where Ca^2+^-mediated signaling plays a crucial role. Pathological cardiac remodeling is recognized as an early feature of the diseased heart and most often transitions to HF with the critical involvement of Ca^2+^-dependent proteins, most notably CaMKII and CaN [84,85,86]. Hence, substantial efforts have been made in the last years to prevent or halt disease progression by restoring impaired cellular Ca^2+^ handling and Ca^2+^-dependent signaling pathways [7,87]. Common triggers of cardiac remodeling involve neurohumoral stimulation, pressure and volume overload and electrical abnormalities leading to arrhythmogenesis. The majority of conventional therapeutic strategies, e.g., β-blockers or angiotensin-converting enzyme (ACE) inhibitors, are directed towards alleviating the effects of neurohormonal overactivation. Newer attempts in treating cardiac remodeling and HF have focused on CaMKII and CaN as therapeutic targets [88,89,90,91], however with no clinical success, which is commonly attributed to the lack of specificity and selectivity of the drugs applied [92]. Similarly, gene therapy approaches to restore physiological SR Ca^2+^ reuptake, although promising in preclinical models [93], have failed to produce results in HF patients [94,95]. Encouraging results, however, were published very recently by Lebek et al. showing that CRISPR-Cas9 base editing to ablate the oxidative activation sites of CaMKIIδ was effective in protecting the heart from ischemia-reperfusion damage in mouse models [96]. 

Therefore, in order to identify novel and relevant therapeutic targets, strategies and/or candidate pharmacotherapeutics against cardiac remodeling, an improvement of the current knowledge base regarding spatio-temporal Ca^2+^ signaling regulation and interplay is imperative. 

#### 4.1.1. Early Cardiac Remodeling

Structural and functional cardiomyocyte remodeling in end-stage HF has been assessed extensively during the last decades, and disturbed Ca^2+^ homeostasis is now considered a hallmark of the terminal disease phenotype. Growing evidence, however, suggests that disturbed Ca^2+^ handling, especially in the cell nucleus, may be an early event in myocardial remodeling and that it may be causally involved in the development of hypertrophy and HF.

We provided the first direct evidence linking structural and functional NE changes to altered nucleoplasmic Ca^2+^ handling in early cardiac remodeling [28]. We observed progressive decline in NE invagination density, increases in nuclei sizes and changes in Ca^2+^-regulatory protein expression patterns on and around the NE following 1 week of transverse aortic constriction (TAC). Decreased NE invagination density harbors important consequences on nuclear Ca^2+^ cycling as it results in slower nuclear CaT propagation (partly due to fewer and less deeply located NPCs) and reduced expression of Ca^2+^ handling proteins such as SERCA. Most importantly, alterations in nuclear CaTs occurred well before cytoplasmic CaTs were measurably affected and they could activate the nuclear CaMKII-HDAC4 axis [28]. In agreement with these findings, Shimojima et al. reported an increase in nuclear area, decrease in number of nuclear invaginations and increase in half-decay time of the nuclear CaTs in neonatal rat ventricular cardiomyocytes in response to potent hypertrophic stimuli such as Ang II, ET-1 and PE [97].

Our more recent work provided further insights into the specifics of early remodeling with respect to nuclear CaTs and the localization of active CaMKIIδC [25]. At the very early phase of cardiomyocyte response to TAC—day 5 post-intervention—we observed enhanced cytoplasmic and nucleoplasmic CaT amplitudes and faster [Ca^2+^]_cyto_ and [Ca^2+^]_nuc_ kinetics. The changes in nucleoplasmic CaTs were more prominent than the cytoplasmic ones, and associated with accumulation of activated CaMKIIδC on the NE. This initial phenotype of preferentially sped up nuclear CaTs prevented the rise of diastolic [Ca^2+^]_nuc_ and the activation of hypertrophic signaling pathways mediated by IL6R. However, such adaptative response was of limited duration only before transitioning into maladaptive changes, first in the nucleus and then in the cytoplasm as well [25,98]. 

Transient enhancement of CaTs, especially in the nucleus, was also observed in young spontaneously hypertensive rats, a well-characterized model of ventricular hypertrophy due to systemic hypertension. It was associated with highly increased SERCA activity and resulted in enhanced nuclear Ca^2+^ signaling via the CaMKIIδ-HDAC4 axis [80].

Along with changes in nuclear CaTs, changes in active receptor-mediated regulation of nuclear Ca^2+^ handling have also been documented [28,99]. In mice that developed hypertrophy due to chronic Ang II infusion, significant increases in IP_3_R2 expression were found at the NE and associated with increased [Ca^2+^]_nuc_ [44]. Strikingly, elevated nuclear Ca^2+^ levels persisted even 3 weeks after removal of the Ang II stimulus and were sufficient to keep CaN active, thereby further increasing IP_3_R2 expression. The proposed hypothesis is that elevation of [Ca^2+^]_nuc_ through newly expressed IP_3_Rs would continue promoting a vicious cycle of CaN-NFAT pro-hypertrophic transcription [44].

#### 4.1.2. Late Cardiac Remodeling

When pathological changes in cellular Ca^2+^ handling, gene expression, structure and function persist, they gradually start manifesting as cardiac dysfunction at organ level and eventually progress to the full-blown HF phenotype [82]. Simultaneously, Ca^2+^-dependent signaling via the CaN-NFAT-GATA4 and CaMKII-HDAC-MEF2 axes remains critically activated, promoting pathological remodeling by altering the phosphorylation status of respective upstream regulators and initiating their nuclear translocation [7,28,80].

**Figure 2:**
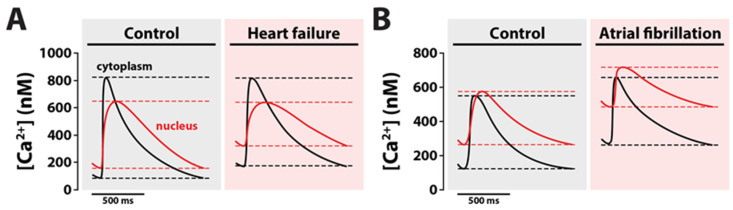
Averaged original recordings of nucleoplasmic (red) and cytoplasmic (black) Ca^2+^ transients from isolated cardiomyocytes. (**A**) Averaged nucleoplasmic (red) and cytoplasmic (black) Ca^2+^ transients of 1 Hz-stimulated isolated cardiomyocytes from sham-operated control mice (grey panel) and mice 7 weeks after transverse aortic constriction (TAC) intervention to induce pressure-overload mediated hypertrophy and heart failure (pink panel). TAC increased both cytoplasmic and nuclear diastolic Ca^2+^ concentration ([Ca^2+^]_cyto_ and [Ca^2+^]_nuc_, respectively), decreased Ca^2+^ transient amplitude and prolonged time to peak and time from peak [Ca^2+^] to 50% decline. (**B**) Averaged nucleoplasmic (red) and cytoplasmic (black) Ca^2+^ transients of 1 Hz-stimulated isolated canine atrial cardiomyocytes from healthy controls (grey panel) and dogs subject to 1 week of atrial fibrillation pacing protocol (600 bpm) prior isolation (pink panel). [Ca^2+^]_nuc_ averages 55% higher in AF vs. CTL with reduced nucleoplasmic Ca^2+^ transient amplitude and significantly increased time to peak and time from peak [Ca^2+^]_nuc_ to 50% decline. Adapted with permission from Refs. [28,107]. 2023, Wolters Kluwer Health, Inc., Philadelphia, USA.

We specifically investigated consequences of long-term cardiac remodeling due to pressure overload on nuclear Ca^2+^ homeostasis and showed that 6–7 weeks post-TAC, diastolic [Ca^2+^] rose, CaT amplitude decreased and the rate of [Ca^2+^] decline slowed down in both cytosol and nucleus [25,28] (Figure 2A). This may be—at least in part—driven by impaired SERCA function and increased RyR2s open probability [100], as well as significant reduction in SERCA expression versus increases in IP_3_R2 levels in nuclear protein fractions from failing human myocytes [28]. At the same time, persistent CaMKII overactivation was visible and abolished by CaMKII inhibitor KN-93. Consistent with chronic CaMKII activation being a causal factor in the development of the disturbed Ca^2+^ phenotype, cardiomyocytes isolated from 11–13 week old CaMKIIδC overexpressing transgenic mice showed signs and symptoms of HF and were characterized by highly elevated diastolic [Ca^2+^] and slowed CaT kinetics in both subcellular compartments [25].

Importantly, we could determine distinct spatio-temporal profiles of CaMKII activation in early and late remodeling. Namely, in contrast to CaMKII accumulation on the NE observed in early remodeling which showed to be protective, in cardiomyocytes isolated from late TAC (6–7 weeks after surgery), CaMKIIδC translocated into the nucleus and promoted HDAC4 export [25]. Indeed, CaMKII mobility and its nuclear import are potentiated by increased intracellular Ca^2+^ levels and CaMKII autophosphorylation [101]. Translation of these findings into human myocardium revealed a large increase of CaMKIIδC expression in failing hearts versus non-failing controls with an especially strong rise in CaMKIIδC in the nuclear fraction. This is consistent with the idea that once CaMKIIδC starts accumulating inside the nucleus, it drives maladaptive transcriptional effects and eccentric hypertrophy [25]. Of note, prolonged CaMKIIδC activation is also observed in response to neurohormonal stimulation via ET-1, Ang II or α-/β-AR agonists (reviewed in [102]); however, the specific contribution of nuclear Ca^2+^ disbalance in these settings remains to be investigated.

In our latest work, we could demonstrate that defective mitochondrial Ca^2+^ uptake typically observed in cardiac remodeling and HF also contributes to increased diastolic [Ca^2+^]_nuc_ in cardiomyocytes [21]. In particular, we demonstrated that perinuclear mitochondria from failing cardiomyocytes are more susceptible to depolarization of mitochondrial membrane potential, reactive oxygen species generation and impairment in Ca^2+^ uptake compared with intrafibrillar mitochondria at baseline and under physiological stress protocol. This may partially explain a disproportionate rise in [Ca^2+^]_nuc_ compared to [Ca^2+^]_cyto_ in failing cardiomyocytes at increased stimulation frequencies. On the other hand, upregulation of the mitochondrial calcium uniporter (MCU) under stress conditions elicits protective effects against cardiomyocyte death from Ca^2+^ overload [103]. MCU upregulation was capable of maintaining intracellular Ca^2+^ and energy homeostasis and thereby limited cardiac hypertrophy and dysfunction in response to chronic β-AR stimulation. Mechanistically, β-AR stimulation activates the nuclear CaMKIIδ variant, CaMKIIδB, which upregulates MCU gene transcription via the phosphorylation of CREB. Therefore, targeting the CaMKIIδB-CREB-MCU axis may present a novel approach in alleviating chronic stress-induced alterations in Ca^2+^ cycling and pathological cardiac remodeling [103]. Furthermore, Liu et al. recently showed in a guinea pig model of HF and sudden cardiac death that moderate MCU overexpression even has beneficial effects that persist weeks after the onset of HF and potently reverses the course of cardiac decompensation and arrhythmogenesis [104]. Ultimately, studying the interplay between (perinuclear) mitochondria, nuclear Ca^2+^ and downstream transcriptional regulation presents a promising and exciting field for future research and the development of new therapeutic options.

Finally, several reports describe NPC rearrangements and changes in nuclear trafficking in animal models and human failing cardiomyocytes and indicate their potential for therapeutic intervention [105,106]. By examining the properties of nuclear importins and exportins, such as expression and transportation magnitude and rate, Chanine et al. have found a reduction in nuclear import versus export, as well as changes in cell and nuclear size, reversible by leptomycin B [105]. We can speculate that changes in NE structure and cargo transport mechanisms may also translate into changes in nuclear Ca^2+^ handling and its downstream signaling; however, the experimental evidence is yet to be provided.

The complex dysregulation of nuclear Ca^2+^ homeostasis during all stages of cardiac remodeling, manifested through transcriptional and functional alterations, highlights the importance of [Ca^2+^]_nuc_ as a clinically relevant parameter and potential therapeutic target for HF patients.

### 4.2. Nuclear Ca^2+^ in Atrial Fibrillation

While ventricular and atrial cardiomyocytes share functional and structural commonalities in the form of ion handling and ECC, there are distinct tissue-specific differences in distribution of ion channel expression and thus electrophysiological properties. AF is the most common sustained cardiac arrhythmia with a lifetime risk of around 20% [108] and substantial health consequences. The propensity towards ectopic firing and re-entry, two key AF features, is aggravated by ion channel dysfunction, Ca^2+^ handling abnormalities, structural remodeling and autonomic neural dysfunction [109]. An increase in global average life expectancy, survival rate and risk factor exposure, coupled with the absence of optimal treatment strategies and continuous rise of AF incidence and prevalence, fundamentally underlie the high socio-economic burden of AF [110]. Similar to HF, most of the current research efforts are devoted to unraveling the mechanisms of global Ca^2+^ handling in AF and fail to consider [Ca^2+^]_nuc_ and its downstream transcriptional targets as contributors to atrial arrhythmogenesis and contractile dysfunction.

The first study that specifically focused on nuclear Ca^2+^ alterations in atrial cardiomyocytes during AF was published only recently [107]. Isolated atrial cardiomyocytes from AF mongrel dogs showed nuclear enlargement and loss of NE invaginations, which was associated with reduced amplitude of nuclear CaTs and higher diastolic [Ca^2+^]_nuc_ in AF samples, mediated by slower nuclear [Ca^2+^] decay kinetics (Figure 2B). Furthermore, even resting [Ca^2+^]_nuc_ averaged 55% higher in AF vs. CTL cardiomyocytes, indicating a true accumulation of nuclear Ca^2+^ in AF. Expression patterns of Ca^2+^-regulating proteins were disturbed in AF, with the most striking increase in IP_3_R levels. Namely, IP_3_R1 expression increased upon AF on the NE and in non-nuclear compartments, while the smaller increase in IP_3_R2 expression was restricted to non-nuclear compartments. Importantly, IP_3_R-dependent Ca^2+^ mobilization from the NE was identified as an important driver of the observed changes in nuclear Ca^2+^ homeostasis, as IP_3_R inhibition resulted in a substantial return of nucleoplasmic CaTs to near control levels. Further, siRNA-mediated IP_3_R-knockdown in canine atrial cardiomyocytes largely diminished diastolic nuclear Ca^2+^ increases caused by in vitro tachypacing [107]. IP_3_R1 dysregulation emerged earlier as a key upstream factor decreasing LTCC current, a functional hallmark of AF [111]. Accumulation of [Ca^2+^]_nuc_ correlated with increased nuclear CaMKII autophosphorylation and HDAC4 export, which was reversed upon IP_3_R-knockdown or CaMKII inhibition [107]. Finally, dysregulation of IP_3_R1 and thus nuclear Ca^2+^ handling in AF was accompanied with strong downregulation of micro-RNA 26, which may represent an exciting new target for future therapeutic interventions [107].

The observed changes in AF also harbor detrimental consequences for ventricular function and cardiomyocyte Ca^2+^ homeostasis, as evidenced in human left ventricular tissue from AF patients and induced pluripotent stem cell (iPSC)-derived cardiomyocytes under AF stimulation protocol [112]. In agreement with previously discussed studies, disturbed Ca^2+^ handling due to AF-simulating pacing protocol in iPSC-CMs induced CaMKII activation [112], which is likely to further promote a vicious cycle of Ca^2+^ dysregulation [25]. Thus, restoring (nuclear) Ca^2+^ handling in AF emerges as an exciting new area of research and it may offer strategies to prevent the potential development of both atrial and ventricular dysfunction in AF patients.

### 4.3. Nuclear Ca^2+^ in Familial Cardiomyopathy

Familial cardiomyopathy accounts for 30% to 50% of all dilated cardiomyopathy cases [113]. Among the genetic causes of familial cardiomyopathy numerous mutations in the structural proteins found in the nucleus, such as lamins and emerin have been identified [114,115,116]. These proteins are important for maintaining NE integrity, chromatin organization and regulation of gene transcription [116]. Mutations in these genes cause arrhythmogenic behavior, which was previously tied to disturbed Ca^2+^ handling and CaMKII [117,118]. Importantly, recent work showed that siRNA-mediated knockdown of emerin encoding gene emd—ultimately causing X-linked Emery-Dreifuss muscular dystrophy (X-EDMD)—was associated with nuclear Ca^2+^ dysregulation in neonatal rat ventricular cardiomyocytes. Likely provoked by the observed decrease in nuclear invagination incidence and thus disturbed Ca^2+^ pump back function into the NE, increased half-decay time of the nuclear CaT may contribute to the initiation of hypertrophic gene program activation and cardiac remodeling through prolonged high [Ca^2+^]_nuc_ [97].

Disturbed cytosolic Ca^2+^ handling was studied more extensively and it was documented in cardiomyocytes with mutations in sarcomeric and ion handling genes such as TNNT (encoding troponin; [119]) and PLN (encoding PLB; [120]), while mutations in the splicing factor RNA binding motif 20 (RBM20), which controls alternate splicing of CaMKIIδ and titin, lead to an increase in cytoplasmic diastolic Ca^2+^, peak transient amplitude, increased SR load and increased frequency of spontaneous Ca^2+^ release in the cytosol [121]. Indeed, due to specific PLB accumulation around the nucleus [19,35] and CaMKIIδ translocation to the nuclear compartment over the course of cardiac remodeling [25,101], it is tempting to speculate that these mutations would have even stronger and/or earlier effects on nucleoplasmic Ca^2+^ levels and their regulation—yet experimental evidence needs to be provided in future.

## 5. Nuclear Ca^2+^: A Practical Approach

Reliable and accurate quantification of subcellular Ca^2+^ signals in cardiomyocytes is essential for assessing and understanding compartmentalized Ca^2+^ fluxes and their roles in ECC and ETC [122]. For successful experimental outcomes, an important aspect to consider is the choice of appropriate Ca^2+^ indicator and experimental model for acquiring signals in the subcellular compartment of interest.

### 5.1. Ca^2+^ Indicators

Detection of [Ca^2+^] changes in different cellular spaces is directly linked to Ca^2+^ binding affinities of fluorescent Ca^2+^ dyes, reflected by the dissociation constant (K_d_). K_d_ allows an estimate of the detectable [Ca^2+^] range and should generally be near the midpoint of the expected [Ca^2+^] fluctuations [123]. Thus, low affinity Ca^2+^ indicators (e.g., Fluo-5N, Mag-fluo 4) are typically used for the visualization of [Ca^2+^] changes in the SR or NE and high affinity Ca^2+^ indicators (e.g., Fluo-3, Fluo-4, Fluo-8) for measuring changes in the cytosolic and nucleoplasmic free [Ca^2+^] [124,125,126,127]. Although Fluo-3 and Fluo-4 are the most commonly used Ca^2+^ indicators in cardiomyocytes, the emerging chemical indicator Calbryte-520 (or Cal-520) showed superior performance in intracellular retention, brightness and fluorescence intensity in CHO-K1 cell line [128]. Cal-520 was also found suitable for drug-screening purposes in iPSC-CM [129]; however, analyses were limited to cytosolic CaTs only. 

Despite being an invaluable research tool since their discovery, chemical fluorescent Ca^2+^ indicators have several technical shortcomings: (1) their Ca^2+^ binding affinities and fluorescent properties vary between cytosol and nucleus, (2) they may be differentially sequestered into intracellular organelles or (3) they may leak from the cytoplasm to the extracellular medium via sarcolemmal anion transporters [32,130]. To overcome these challenges, substantial efforts have been made to transform raw fluorescence signals into calibrated [Ca^2+^] by determining in situ calibration curves in different types of cardiomyocytes isolated from different experimental animal models. Such curves take into account the effects of a particular cellular environment on indicator properties and allow for a direct comparison between [Ca^2+^] in different subcellular spaces [32,44,80,107,125]. An additional hurdle to consider is that cardiac remodeling processes may impact K_d_, meaning that in some cases, signal calibration has to be performed independently for control and experimental groups [107]. Apart from the technical shortcomings one should keep in mind that Ca^2+^ fluorophores exhibit detrimental physiological effects on sodium-potassium ATPase (NKA) activity, cell viability and metabolic status [131], which can further complicate their use.

In contrast, genetically encoded fluorescent Ca^2+^ indicators (GECIs) interfere only minimally with NKA activity [131] and they have a big advantage with regard to their specific targeting to cellular compartments or organelles of interest via specific promoters and targeting sequences. GCaMP, one of the most successful and popular type of GECIs, has been continuously improved and updated to ultimately provide rapid response kinetics and powerful signal-to-noise ratios in subcellular Ca^2+^ imaging [132,133]. However, the CaM component in GCaMPs serving as the Ca^2+^ sensor element raised concerns regarding its use, as it was found to interfere with the gating of LTCCs in neurons leading to chronic nuclear Ca^2+^ accumulation and transcription dysregulation [134]. Similar effects on cardiac LTCCs have not been reported but may be prevented by modulating the CaM motif [134]. Another member of the GCaMP family, GCaMP6s, was evaluated as knockin in hPSC-CMs and successfully visualized isoprenaline-induced alterations in CaT kinetics [135]. Addition of a nuclear localization sequence, as in the case of jGCaMP7, allows highly efficient nuclear-specific targeting and thus real-time nuclear CaT acquisition as demonstrated in a mouse fibroblast line [136]. 

Promising progress has been recently made by combining newly designed MaPCa dyes—highly permeable rhodamine-based Ca^2+^ indicators of varying affinities and colors—with HaloTag fusion proteins targeted to specific subcellular localizations and compatible with both fluorescence and bioluminescence readouts [137]. In the first Ca^2+^ imaging experiments, AM esters of the MaPCa indicators were applied to co-cultures of 293 cells stably expressing a nuclear-localized HaloTag and nonexpressing 293 cells, demonstrating great power of the HaloTag expressing cells in detecting nuclear Ca^2+^ fluxes with excellent signal-to-background ratios [137]. Importantly, these indicators can be excited in the far-red range, thus enabling a more precise and detailed analysis of the Ca^2+^ dynamics in cardiomyocytes with special regard to the inter-compartmental crosstalk.

### 5.2. Emerging Models, Tools and Future Perspectives

Nuclear Ca^2+^ fluxes have been mostly monitored in cardiomyocytes from experimental animal models. While these models are necessary for in vivo and in vitro analyses of cardiac function in cardiac (patho)physiology, they differ fundamentally from humans. As such, results derived from preclinical studies, particularly in rodents, are not always transposable to humans. On the other hand, human cardiac tissue is either obtained from organ donors or as surgical biopsies [28,107,138,139], thus sample availability is relatively limited. With an increasing need for more “translatable” but at the same time readily accessible in vitro models, human induced pluripotent stem cell-derived cardiomyocytes (hiPSC-CMs) have quickly proven invaluable. They reproduce many of the human cardiomyocyte characteristics such as Ca^2+^ cycling, contractility and response to exogenous stimuli, key sarcomeric proteins and Ca^2+^ signaling protein expression [140,141,142,143,144,145,146] and are—importantly—emerging as a tool for studying nuclear Ca^2+^ signals [147]. Furthermore, hiPSC-CMs can be derived from patients with preexisting genetic mutations influencing nuclear structure and consequently Ca^2+^ handling, such as lamin A/C cardiomyopathy (LMNA-DCM) [148], X-EDMD [149], but also mutations in CaMKIIδ splicing factor RBM20 [121].

The remaining downside of using hiPSC-CMs, however, is their incomplete maturation. To overcome this, advances in tissue engineering have enabled the development of novel two- or three-dimensional cardiovascular tissue models, namely cardiac organoids/microtissues and engineered heart tissue with superior cardiomyocyte maturation phenotypes [150,151]. However, detection of nuclear CaTs in such models is limited due to lack of standard digestion protocols that will consistently recover single cardiomyocytes from 3D structures with a high cellular yield and small penetration depth of confocal imaging systems. To surmount the challenges, Richards et al. developed a two-component imaging system able to track single cell CaTs from GCaMP6 labeled hiPSC-CMs within a microtissue model of cardiac toxicity. They took advantage of the increased scanning depth of two-photon excitation microscopy and the increased resolution of light-sheet microscopy systems equipped with a high-speed camera to record subcellular Ca^2+^ events [152]. Despite the current limitations, improving the isolation of single cardiomyocytes from cardiac organoids, as well as advancing technology for visualization of nuclear CaTs in intact microtissues holds a great promise for future work. 

To our knowledge, two genetically encoded Ca^2+^ chelating proteins for selectively modulating (peri)nuclear [Ca^2+^] have been successfully used in isolated cardiomyocytes in the past [10,153]. In work by Higazi et al., nuclear Ca^2+^ was specifically buffered using a nuclear-targeted, red fluorescent protein-tagged form of the neuronal Ca^2+^ binding protein calbindin, demonstrating that increased nuclear Ca^2+^ levels are required for the induction of atrial natriuretic factor expression [153]. Turcotte et al. used mCherry-tagged parvalbumin β-nesprin fusion protein designed to buffer Ca^2+^ in the perinuclear space and showed that buffering perinuclear Ca^2+^ completely inhibits isoprenaline-induced perinuclear CaN activation in both neonatal and adult myocytes [10]. Further optimization and/or knockin of such nuclear Ca^2+^ regulatory proteins can be of great value for studying nucleus-restricted Ca^2+^ dynamics.

## 6. Conclusions

In this review, we demonstrate that nuclear Ca^2+^ handling—although not as extensively studied as an independent parameter of cardiac remodeling—significantly complements the knowledge on cytoplasmic Ca^2+^ regulation and alterations, and it augments the likelihood of mitigating pathological cardiac remodeling in the coming years. Future technological breakthroughs in the field of subcellular Ca^2+^ quantification, cardiac disease modeling and small molecule generation will surely help to better describe remodeling mechanisms and prevent or even halt cardiac disease progression. Understanding nuclear Ca^2+^ regulation will undoubtedly play an important role in these efforts, as Ca^2+^ cycling in the nucleus may be the “mastermind” behind the initiation and progression of various cardiac diseases [154].

## Data Availability

No new data were created or analyzed in this study. Data sharing is not applicable to this article.
